# Trillin protects against doxorubicin-induced cardiotoxicity through regulating Nrf2/HO-1 signaling pathway

**DOI:** 10.1371/journal.pone.0321546

**Published:** 2025-04-08

**Authors:** Xinyi Yang, Sili Liu, Miyan Liu, Didong Lou, Wenjun Zou, Xiaofen Li

**Affiliations:** 1 Guizhou University of Traditional Chinese Medicine, Guiyang, China; 2 Chengdu University of Traditional Chinese Medicine, Chengdu, China; BRAC University, BANGLADESH

## Abstract

Doxorubicin (DOX) is widely employed in anticancer therapy, but its clinical application is constrained by its cardiotoxic effects. Trillin, a bioactive compound derived from *Trillium tschonoskii* Maxim., has been identified as a natural antioxidant possessing cardioprotective properties. This study aimed to ascertain whether trillin can protect against DOX-induced cardiotoxicity (DIC) through its inherent antioxidant capabilities. In vivo studies, C57BL/6 mice were administered DOX (5 mg/kg i.p.) via intraperitoneal injection once weekly for a total of five consecutive weeks and received trillin (25, 50 and 100 mg/kg i.g.) through intragastric administration once daily for six weeks. In vitro studies, H9c2 cardiomyocytes were utilized to verify the protective efficacy of trillin (0.5, 1 and 2 μM) against DIC. Trillin significantly mitigated DOX-induced myocardial damage, which encompassed improvements in left ventricular function, reductions in serum cardiac enzymes levels, and diminution of heart cell vacuolation. Moreover, trillin effectively attenuated DIC while preserving the anticancer efficacy of DOX. Trillin also alleviated oxidative injury by elevating levels of SOD and GSH and reducing MDA levels. Additionally, trillin restored the expression of Nrf2 and HO-1 in mouse hearts and H9c2 cardiomyocytes treated with DOX. Trillin safeguarded against DIC by inhibiting oxidative stress via upregulation of the Nrf2/HO-1 pathway. These findings furnish evidence suggesting trillin may serve as a therapeutic agent for the prevention of DIC.

## 1 Introduction

Cancer is a destructive disease worldwide that is increasing in incidence and mortality. Doxorubicin (DOX) is an effective anthracycline chemotherapeutic drug used to treat a wide range of solid tumors and malignancies [1,2]. However, the clinical application of DOX has been severely limited due to its cumulative and dose-dependent cardiotoxicity [3]. According to published studies, long-term treatment with DOX can lead to cardiomyopathy, most notably characterized by a reduction in left ventricular ejection fraction (LVEF), an increase in ventricular internal diameter, and subsequent severe congestive heart failure [4,5]. Among patients receiving 400, 550, and 700 mg/m^2^ of DOX, the incidence rates of heart failure are 5%, 26%, and 48%, respectively [6,7]. In addition, among child cancer survivors who have undergone DOX treatment, echocardiographic abnormalities are detected in up to 50% of the patients, and approximately 10% of them develop cardiomyopathy [8,9].

Clinical strategies for preventing cardiotoxicity due to anthracycline drugs include reducing the cumulative dosage, continuous infusion, and using less toxic analogues [10]. However, reducing the cumulative dosage of DOX to 550 mg/m^2^ or using analogues of DOX can significantly impair the antitumor effects. Moreover, the risk of inducing cardiomyopathy is not reduced [11]. Dexrazoxane is the only drug approved by the US Food and Drug Administration (FDA) for treating cardiotoxicity caused by anthracyclines. However, it can exacerbate chemotherapy-induced myelosuppression and has been shown to increase the risk of secondary malignancy [12,13]. So, it’s crucial to develop therapeutic agents that effectively treat DOX-induced cardiotoxicity without exhibiting negative effects or reducing the anticancer efficacy of DOX.

Numerous studies have shown that the underlying mechanism of DIC is complex and multifactorial, involving mitochondrial damage, oxidative stress, pyroptosis, ferroptosis and apoptosis [14]. It is worth noting that during the process of DOX-induced oxidative stress, free radicals formed by any of these mechanisms continue to damage various cellular components, including nucleic acids, and proteins. This leads to mitochondrial dysfunction, and subsequently activates cytotoxic signaling pathways, causing damage to myocardial tissue [15]. Therefore, oxidative stress is a key factor in DIC. The expression levels of endogenous antioxidant enzymes in the heart are relatively low, which makes the heart more susceptible to DOX-induced damage [16]. Evidence has indicated that DOX induces myocardial oxidative stress by regulating levels of SOD, and GSH, MDA [17]. A mediator of responses to oxidative stress, namely, Nrf2 is significantly downregulated in DIC, leading to insufficient expression of antioxidant enzymes [18]. Research has shown that the activation of Nrf2 reduces DOX-induced oxidative effects on cardiomyocytes. In addition, the natural compound glycyrrhetinic acid alleviates DIC by activating Nrf2 [19]. Activation of the Nrf2 pathway may be effective in alleviating oxidative stress and cardiac injury.

**Fig 1:**
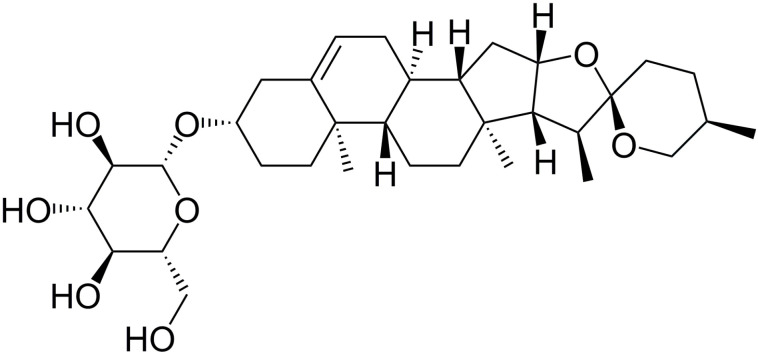
Molecular structure of trillin.

Trillin (Fig 1) is a steroidal saponin that is abundant in herbal medicinal plants such as *Trillium tschonoskii* Maxim., *Dioscorea nipponica* Makino, and *Dioscorea panthaica* Prain & Burkill. Its chemical structure is diosgenin-3-O-β-D-glucopyranoside. Trillin has multiple pharmacological effects, including antitumor, antioxidative, free radical scavenging, and immunomodulatory effects [20–23]. Previous studies have shown that trillin can inhibit oxidative stress in tissues by activating the Nrf2 pathway, thereby protecting against spinal cord injury in rats [24]. Moreover, trillin has been reported to protect against D-galactose-induced myocardial injury by inhibiting myocardial mitochondrial autophagy [25]. We speculated that trillin may be an effective candidate drug for the treatment of DIC. However, the potential effects of trillin in treating DIC and their underlying mechanisms have not been thoroughly investigated. So, we investigated the possible cardioprotective effects of trillin through in vivo and in vitro experiments. Moreover, we also studied whether trillin can protect against DIC by alleviating oxidative stress via regulation of the Nrf2 pathway.

## 2 Materials and methods

### 2.1 Drugs and reagents

Trillin (purity ≥  98%, CAS No. 14144-06-0, Lot No. MUST-23110713) purchased from Chengdu MUST Biotechnology Co., Ltd (Chengdu, China). DOX and Nrf2 inhibitor ML385 were purchased from Selleck (Houston, USA). Creatine Kinase-MB Isoenzyme (CK-MB), lactate dehydrogenase (LDH), and aspartate aminotransferase (AST) detection kits were purchased from Mindray (Shenzhen, China). Assay kits for CAT, GSH, SOD, and MDA were purchased from Elabscience Biotechnology Co.,Ltd (Wuhan, China). Anti-Nrf2 and HO-1 antibodies was purchased from Cell Signaling Technology (Boston, USA).

### 2.2 Animals and treatment

The male C57BL/6J mice (23 - 25 g, eight - week - old) were purchased from Si Pei Fu Biological Technology Co., Ltd. (Beijing, China). The animals were maintained in a room at a temperature of 22 ± 2°C under a 12 - hour dark/light cycle. In the experiment, the mice were housed in a specific pathogen - free (SPF) grade barrier environment. All animal experimental surgery were anesthetized with isoflurane gas to relieve pain. Then, blood samples were collected by enucleating their eyeballs, and the mice were euthanized by cervical dislocation. All animal study protocols were approved by the ethics committee of Guizhou University of Traditional Chinese Medicine (approval no: TCM-2023-002), and performed according to the National Institute of Health Guide for the Care and Use of Laboratory Animals.

In the preliminary experiments, we evaluated the effects of Trillin at doses of 10, 15, 25, 50, 100, and 150 mg/kg on echocardiography (LVEF) and myocardial enzymes (CK-MB, LDH) in DIC mice. The results indicated that Trillin at doses of 25, 50 and 100 mg/kg significantly improved echocardiographic parameters and myocardial enzyme levels in DIC mice. Based on these findings, we selected these three doses (25, 50, and 100 mg/kg) for further investigation in the formal experimental phase.

The mice were divided into five groups: Control; DOX; DOX+Trillin (25 mg/kg); DOX+Trillin (50 mg/kg); DOX+Trillin (100 mg/kg). The DOX+Trillin groups were pretreated with trillin (25, 50, and 100 mg/kg/day) for one week. Except for the control group, DOX was administered via intraperitoneal injection once a week at a dose of 5 mg/kg for five weeks. Mice in the trillin groups were treated with trillin (25, 50, and100 mg/kg/day) for six consecutive weeks.

### 2.3 Echocardiographic assessment of cardiac functions

At the end of the treatment, two-dimensional short-axis M-mode echocardiography was using the Vevo 3100 system (FUJIFILM VisualSonics, Canada). The mice were anesthetized with isoflurane gas to alleviate pain, and blood samples were collected by enucleating their eyeballs. Then, the mice were euthanized by cervical dislocation. And the parameters of heart rate (HR), LVEF, LVFS, SV, LVIDs and LVIDd were measured in five consecutive cardiac cycles.

### 2.4 Measurement levels of CK-MB, LDH, and AST

The mice were anesthetized with isoflurane gas to alleviate pain, and blood samples were collected by enucleating their eyeballs. Then, the mice were euthanized by cervical dislocation. Blood was centrifuged at 3000 rpm for 10 minutes, and the supernatant was collected. The serum levels of CK-MB, LDH, and AST were measured using an automatic blood biochemical analyzer (Mindray, China).

### 2.5 Measurement of antioxidant enzyme and MDA levels

The levels of CAT, GSH, SOD, and MDA in heart tissue were determined using a biochemical kit (Nanjing Jiancheng, China).

### 2.6 Heart histopathological examination

Cardiac tissues were fixed with 4% paraformaldehyde embedded in paraffin and sectioned at a thickness of 5 μm. The sections were stained with H&E and photographed using a microscope (Leica, Germany).

### 2.7 Cell culture

The rat H9c2 myocardial cells (No. GNR 5), HepG2 cells (hepatoma cells, No. SCSP510) and H460 cells (lung cancer cells, No. SCSP584) were purchased from the National Collection of Authenticated Cell Cultures (Shanghai, China). The 4T1 cells (mammary gland cancer cells, No. KLA068M) were purchased from Kang Lang Biological Technology Co., Ltd (Shanghai, China). All cells were cultured in DMEM supplemented with 10% FBS and 1% penicillin-streptomycin and maintained in a culture incubator containing 5% CO_2_ at 37°C.

### 2.8 Cell viability

H9c2 cells were plated into 96-well microplates (5000 cells/well). After incubated for 24 hours, and treated with trillin (0.25, 0.5, 1, 2, 4, 8, 16, 20 μM). Briefly, cell viability was detected using the Cell Counting Kit-8 (CCK8) reagent kit. The selected concentrations of trillin were also used to treat the cancer cells.

H9c2 cells were digested into a single-cell suspension and mixed thoroughly by pipetting. Next, 10 µ L of the cell suspension was transferred to a hemocytometer for cell counting. Based on the calculation, culture medium was added to the cell suspension to achieve a cell density of 5000 cells per 100 µL. The H9c2 cells were plated into 96-well microplates (5000 cells/well). After incubation for 24 hours, cells were co-treated with DOX (4 μM) and trillin (0.25, 0.5, 1, 2, 4, and 8 μM) for an additional 24 hours. Finally, cell viability was assessed using CCK8 reagent kit. The effects of trillin on the antitumor ability of DOX were evaluated in 4T1, HepG2, and H460 cells. These cells were treated with DOX (4 μM) in the presence or absence of trillin (0.5, 1 and 2 μM) for 24 hours. Cell viability was determined using CCK8 reagent kit as mentioned above.

(Experimental well OD - Negative control well OD) * 100%/ (Blank well OD - Negative control well OD)

### 2.9 Measurement of LDH and cTnT levels

The levels of LDH and cardiac troponin T (cTnT) in H9c2 cells were determined using a biochemical kit and Elisa kit (Elabscience, Wuhan, China), respectively.

### 2.10 Oxidative stress detection

The levels of CAT, GSH, SOD, and MDA in cardiac tissue and H9c2 cells were determined using a biochemical kit (Elabscience, Wuhan, China).

### 2.11 Real-time PCR analysis

Total RNAs were extracted from mouse heart tissue and H9c2 cells using TRIzol reagent and reverse-transcribed to cDNA. Real-time PCR quantification was performed using the CFX96 System (Bio-Rad, USA).

### 2.12 Western blot assay

Total protein content was detected using a BCA kit and separated on 10% SDS-PAGE gel. The separated protein was transferred to nitrocellulose membrane filters, blocked with 5% skim milk in TBST for 2 hours, and incubated with the primary antibody at 4°C overnight, and diluted secondary antibody for 2 hours. Finally, using an ECL substrate, protein bands were imaged on a chemiluminescence imaging analyzer (Tanon, Shanghai, China).

### 2.13 Inhibition of Nrf2 gene expression in cardiomyocytes

Inoculate H9c2 cells in a 96-well plate (10,000 cells/well) and treat them with the Nrf2 inhibitor ML385 (0, 1, 2, 4, and 8 µM) for 24 hours. Extract total RNA from the cells and evaluate Nrf2 expression via RT-qPCR to determine the optimal concentration of ML385 that inhibits Nrf2 expression. Subsequently, H9c2 cells were seeded in a 96-well plate (5,000 cells/well) and cultured for 24 hours. The control group, DOX group (4 µM DOX), DOX +  trillin group (4 µM DOX and 2 µM trillin), and DOX +  trillin +  ML385 group (4 µM DOX, 2 µM trillin, and 1 µM ML385) were established. After 24 hours of cell treatment, the levels of Nrf2 and HO-1 proteins in H9c2 cells were detected by Western blot assay.

### 2.14 Statistical analysis

The data were analyzed using SPSS 24.0 software and expressed as the mean ±  SD. One-way analysis of variance followed by Tukey’s post hoc tests was performed to compare the mean values of multiple groups. Statistical significance was considered if *P* < 0.05 or *P* < 0.01.

## 3 Results

### 3.1 Trillin alleviated DOX-induced myocardial dysfunction in mice

**Fig 2:**
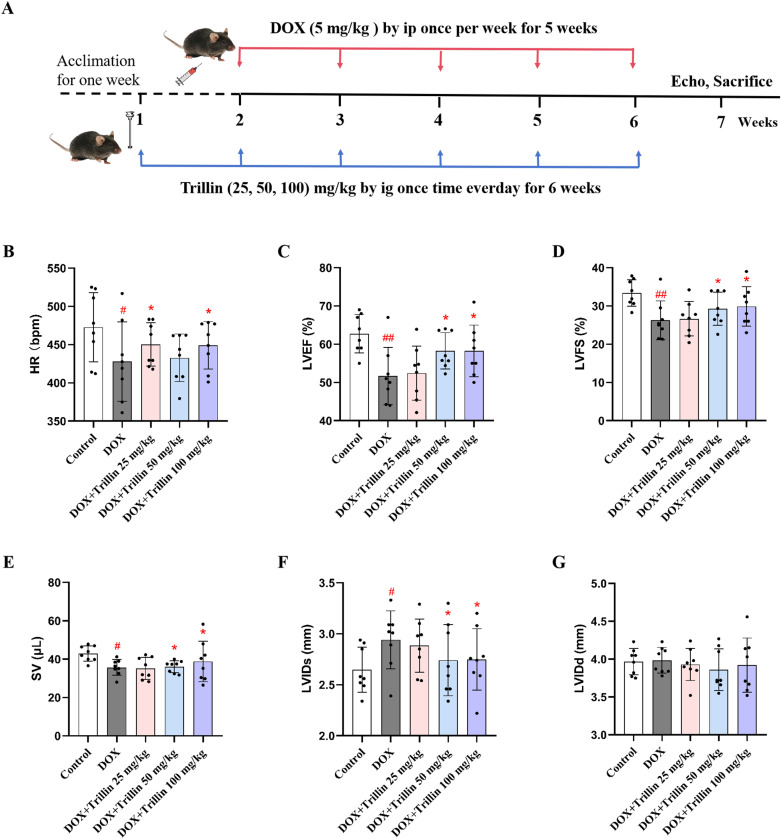
Trillin improved DOX-induced cardiac dysfunction in C57 mice. (A) Schematic protocol for DOX and trillin treatment. (B-G) Echocardiography parameters of HR, LVEF, LVFS, SV, LVIDs, and LVIDd in mice (n = 8). ^#^*P* < 0.05, ^##^*P* < 0.01 vs control; * *P* < 0.05, vs DOX.

Mice were treated with trillin for six weeks. A mouse model of DIC was then established by intravenous injection of DOX once each week for five weeks (Fig 2A). Echocardiography was used to evaluate the protective effect of trillin against DOX-induced left ventricular dysfunction in vivo. After the mice had received a cumulative dose of 25 mg/kg DOX, significant changes were observed in the function and structure of their hearts. As shown in Fig 2B–F, compared with the control group, HR, LVEF, LVFS, and SV significantly decreased in the DOX group, whereas LVIDs significantly increased (*P* < 0.05; *P* < 0.01). As expected, compared with the DOX group, treatment with trillin significantly reduced the changes in HR, LVEF, LVFS, SV, and LVIDs induced by DOX (*P* < 0.05). Surprisingly, no significant change in LVIDd was induced by DOX and trillin (25, 50 and 100 mg/kg) (Fig 2G).

### 3.2 Trillin alleviated DIC in mice

**Fig 3:**
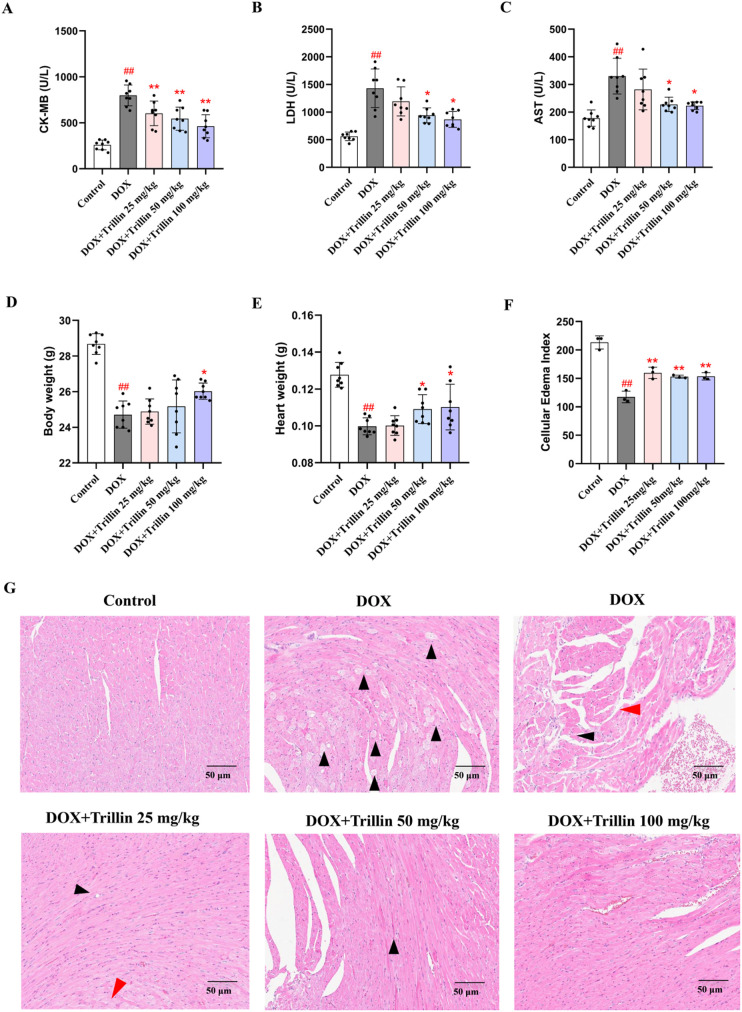
Trillin alleviated DOX-induced cardiac injury in mice. (A-C) Serum levels of CK-MB, LDH, and AST in mice (n = 8). (D-E) Body weight and heart weight of the mice. (F) Cell edema index of myocardial cells in mice. (G) Representative images of H&E staining, (Scale bar =  50 μm). ^##^*P* < 0.01 vs control; * *P* < 0.05, **P < 0.01 vs DOX.

The release of the myocardial enzymes CK-MB, LDH, and AST into serum indicates the extent of myocardial injury [26]. Compared with the control group, DOX significantly increased the levels of CK-MB, LDH, and AST. However, this effect was markedly reduced by treatment with trillin (50 and 100 mg/kg) (Fig 3A–C). Compared with the control group, mice in the DOX group exhibited significant decreases in both body weight and heart weight. Notably, treatment with trillin (100 mg/kg) effectively reversed these DOX-induced changes (Fig 3D–E). Furthermore, HE staining of heart tissue revealed that DOX caused severe histopathological damage, including capillary congestion, vacuolation of myocardial cells, interstitial edema, and loss of myocardial cells. Compared with the DOX group, the cell edema index was significantly reduced in groups treated with trillin (50 and 100 mg/kg) (Fig 3F-G).

### 3.3 Trillin protected against DIC in H9c2 cells

**Fig 4:**
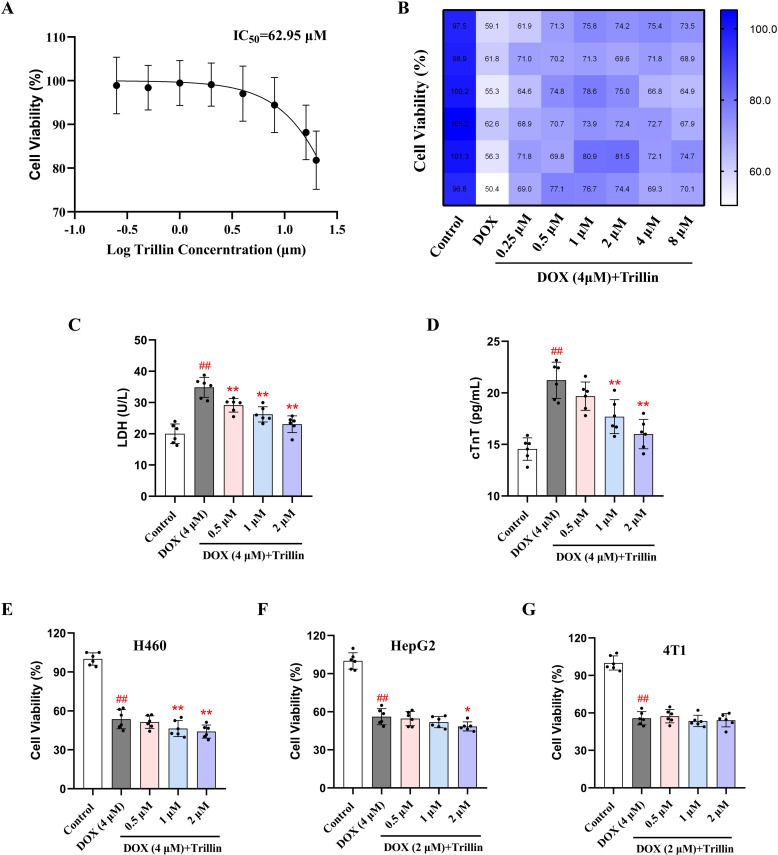
Effects of trillin on H9c2 cells viability and antitumor ability of DOX. (A) Cytotoxicity of trillin on H9c2 cells. (B) H9c2 cells were co-treated with DOX (4 μM) and trillin (0.25, 0.5, 1, 2, 4, 8, 16 and 20 μM) for 24 hours. (C-D) Levels of LDH and cTnT in H9c2 cells. (E-G) H460, HepG2, and 4T1 cells were co-treated with DOX (4 μM) and trillin (0.5, 1 and 2 μM) for 24 hours. (n = 6). ^##^*P* < 0.01 vs control; * *P* < 0.05, **P < 0.01 vs DOX.

As shown in Fig 4A, the half maximal inhibitory concentration (IC_50_) of trillin was determined to be 62.95 μM. Our results showed that treatment with trillin (0.5, 1, and 2 μM) increased cell viability and decreased the leakage of LDH and cTnT in a dose-dependent manner (Fig 4B–D). Furthermore, we assessed the effect of trillin on the antitumor activity of DOX. As indicated in Fig 4E–G, trillin had no effect on the activity of DOX against 4T1 cells. In addition, co-administration of DOX with trillin (2 μM) induced a decrease in the viability of H460 and HepG2 cells compared with DOX alone.

### 3.4 Trillin enhanced antioxidant capacity and inhibited MDA production

**Fig 5:**
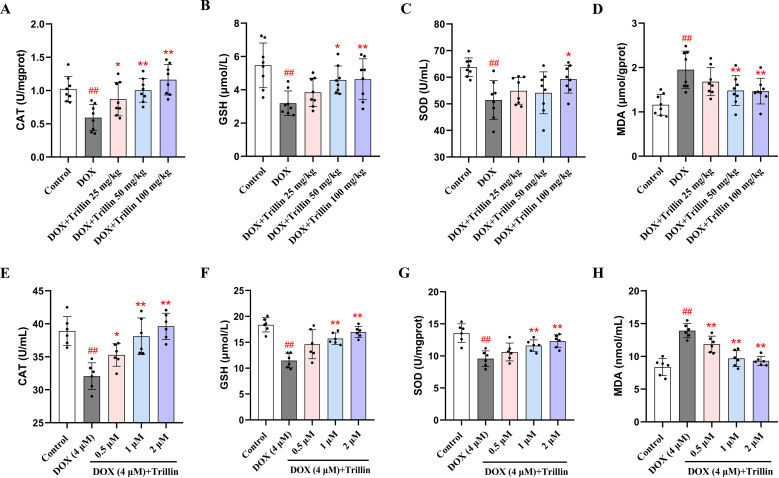
Trillin attenuated DOX-induced oxidative stress. (A-D) CAT, GSH, SOD, and MDA lvels in cardiac tissue of mice. (E-H) CAT, GSH, SOD, and MDA levels in H9c2 cells (n = 6). ^##^*P* < 0.01 vs control; * *P* < 0.05, **P < 0.01 vs DOX.

Oxidative stress is considered a critical cause of DIC [14]. Subsequently, the effects of trillin on the activity of antioxidant enzymes were further investigated. The levels of the antioxidant enzyme CAT, GSH, SOD in mice decreased significantly in the DOX group, while the level of MDA increased (Fig 5A–D). As expected, treatment with trillin mitigated the effects of DOX by increasing the levels of CAT, GSH, SOD, and decreasing the level of MDA. Consistent with the in vivo results, trillin reversed the effects observed in cells treated with DOX alone, including the decrease in CAT, GSH, SOD levels and the increase in MDA levels (Fig 5E–H).

### 3.5 Trillin activated Nrf2 signaling in DOX-treated mice and H9c2 cells

**Fig 6:**
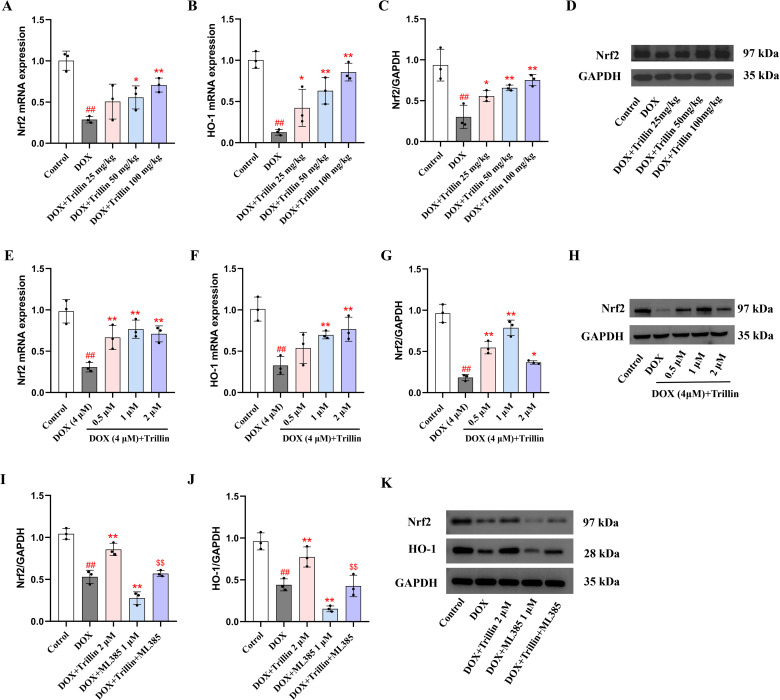
Trillin increased Nrf2 expression. (A-B) The mRNA levels of Nrf2 and HO-1 in mice. (C-D) The protein level of Nrf2 in mice (n = 3). (E-F) The mRNA levels of Nrf2 and HO-1 in H9c2 cells. (G-H) The protein level of Nrf2 in H9c2 cells (n = 3). (I-K) After inhibiting of Nrf2 gene expression in cardiomyocytes, the protein level of Nrf2 in H9c2 cells (n = 3). ^##^*P* < 0.01 vs control; * *P* < 0.05, **P < 0.01 vs DOX; ^$$^*P* < 0.01 vs DOX+ML385.

Nrf2 is a key transcription factor that activates the expression of antioxidant genes [27]. As shown in Fig 6A–D, the mRNA levels of Nrf2 and HO-1 and the protein expression level of Nrf2 were significantly decreased by DOX. However, treatment with trillin reversed these changes caused by DOX in mice. Consistent with the in vitro results, trillin upregulated the mRNA levels of Nrf2 and HO-1 and the protein expression level of Nrf2 in H9c2 cells (Fig 6E–H). The Nrf2 inhibitor ML385 (1 µM) was used to suppress Nrf2 expression in cardiomyocytes, and DOX and trillin treatments were administered simultaneously. The results showed that Trillin (2 µM) reversed the inhibitory effects of DOX on Nrf2 and its downstream target HO-1 expression. Moreever, co-treatment with DOX and ML385 significantly reduced the expression of Nrf2 and its downstream target HO-1. Nevertheless, Trillin significantly alleviated the inhibitory effects of ML385 on Nrf2 and HO-1 proteins. The results indicated that the protective effect of trillin against DIC was related to activation of Nrf2 expression.

## 4 Discussion

In the present study, mice treated with DOX exhibited significant changes in major functional echocardiographic parameters, as well as increased levels of myocardial enzymes. These mice also showed cardiac histological injuries, such as vacuolation of myocardial cells, interstitial edema, and loss of myocardial cells, consistent with previous studies [28]. Our results demonstrated that trillin significantly alleviated DOX-induced cardiotoxicity, as evidenced by improvements in echocardiographic parameters and decreased levels of myocardial enzymes. Trillin also reversed damage to heart tissue. Furthermore, our in vitro experiments showed that trillin effectively increased cell viability and decreased the release of LDH and cTnT induced by DOX in H9c2 cells.

The impairment of the antioxidant defense system is considered a primary mechanism underlying DIC [29,30]. A previous study demonstrated that water spinach, wood apple, and linseed protected against DIC by inhibiting oxidative stress in a rat model [31]. Therefore, upregulating endogenous antioxidant systems is a promising strategy to mitigate DIC. This study primarily aimed to examine trillin’s protective effects against DOX-induced oxidative stress. Our data revealed that DOX significantly decreased CAT, GSH, SOD levels while increasing MDA levels in both mouse cardiac tissue and H9c2 cells. In contrast, trillin treatment significantly reduced oxidative stress induced by DOX. These results indicate that trillin exerts cardioprotective effects by alleviating oxidative stress.

Nrf2, a key transcription factor that regulating cellular redox reactions, lacks antioxidant function on its own. However, when translocated from the cytoplasm to the nucleus, Nrf2 specifically binds to AREs, promoting the expression of the antioxidant gene HO-1 and conferring resistance to oxidative stress [32]. Numerous studies suggest that Nrf2 is a potential target for treating DIC, as it is often inhibited or inactivated in DOX-stimulated models. Activation of the Nrf2/HO-1 pathway can enhance endogenous antioxidant responses in the myocardium, protecting against DIC [33–35]. Our experiments showed that trillin significantly upregulated Nrf2 mRNA and protein expression and increased HO-1 mRNA expression. Collectively, these data suggested that trillin activated the Nrf2/HO-1 pathway to regulate oxidative stress and thus protects against DIC.

Converting drug dosage between animal species is crucial for new drug development. Body surface area (BSA) correlates well with various biological parameters, including oxygen utilization efficiency, energy expenditure, basal metabolism, blood volume, plasma protein levels, and renal function in different mammals [36]. Applying the BSA normalization method, we successfully converted trillin’s dosage from mice to humans. The calculated human equivalent dose of trillin is 4 mg/kg (240 mg for a 60 kg individual), providing a starting point for future clinical trials. However, further toxicological studies are essential to ensure trillin’s safety and efficacy at this and potentially higher doses.

Safe and effective cardioprotective drugs must reduce heart damage without compromising DOX’s anticancer efficacy. Studies have shown that trillin inhibits various cancer cells types and acts as a potent natural anticancer agent [20,37]. We investigated whether trillin affects DOX’s antitumor activity through in vitro studies on a series of cancer cell lines, namely, H460, HepG2, and 4T1 cells. Compared to DOX alone, the combination of trillin and DOX promoted cell death in H460 and HepG2 cells but did not affect 4T1 cells survival. These results suggest that trillin may enhance DOX’s anticancer in specific cancer cells types. The cell-selective properties of trillin in H9c2 cardiomyocytes and tumor cells likely occur in a cell-dependent manner. Future research should focus on elucidating the mechanisms underlying trillin’s selective activation of the Nrf2/HO-1 pathway and its anticancer effects. Additionally, exploring synergistic interactions between trillin and other chemotherapeutic agents could uncover new strategies to improve cancer treatment outcomes while minimizing cardiotoxicity.

## 5 Conclusions

In conclusion, our research demonstrated that trillin effectively mitigates DIC by inhibiting oxidative stress through the activation of the Nrf2/HO-1 pathway. This study highlight trillin as a promising candidate for the development of safe and effective cardioprotective agents against DOX-induced cardiotoxicity. Exploring the potential synergistic interactions between trillin and other chemotherapeutic agents could uncover new therapeutic strategies for improving cancer treatment outcomes while minimizing cardiotoxicity.

## Supporting information

S1 File Western blot. (XLSX)

## Abbreviations

AST: Aspartate aminotransferase
HO-1: Heme oxygenase-1;
BSA: Body surface area
IC_50_: half maximal inhibitory concentration
CAT: Catalase
LDH: Lactate dehydrogenase;
CCK-8: Cell Counting Kit-8;
LVEF: Left ventricular ejection fraction;
CK-MB: Creatine Kinase-MB Isoenzyme
LVFS: Left ventricular fractional shortening
cTnT: Cardiac troponin T
LVIDd: Left ventricular end-diastolic diameter;
DOX: Doxorubicin
LVIDs: Left ventricular end-systolic diameter
DMEM: Dulbecco’s modified Eagle’s medium
HR: Heart rate
DIC: DOX-induced cardiotoxicity
MDA: Malondialdehyde
ELISA: Enzyme linked immunosorbent assay
Nrf2: Nuclear factor erythroid 2-like 2;
FBS: Fetal bovine serum
ROS: Reactive oxygen species;
GSH: Glutathione
SOD: Superoxide dismutase
H&E: Hematoxylin and eosin
SV: Stroke volume.

## References

[1] Zhu L, Lin M. The synthesis of nano-doxorubicin and its anticancer effect. Anticancer Agents Med Chem. 2021;21(18):2466–77. doi: 10.2174/1871520621666201229115612 33372884

[2] Almajidi YQ, Kadhim MM, Alsaikhan F, Turki Jalil A, Hassan Sayyid N, Alexis Ramírez-Coronel A, et al. Doxorubicin-loaded micelles in tumor cell-specific chemotherapy. Environ Res. 2023;227:115722. doi: 10.1016/j.envres.2023.115722 36948284

[3] Herrmann J. Adverse cardiac effects of cancer therapies: cardiotoxicity and arrhythmia. Nat Rev Cardiol. 2020;17(8):474–502. doi: 10.1038/s41569-020-0348-1 32231332 PMC8782611

[4] Zhu M, Chen Y, Cheng L, Li X, Shen Y, Guo G, et al. Calsyntenin-1 promotes doxorubicin-induced dilated cardiomyopathy in rats. Cardiovasc Drugs Ther. 2024;38(2):237–52. doi: 10.1007/s10557-022-07389-x 36350487 PMC10959838

[5] Yun W, Qian L, Yuan R, Xu H. Periplocymarin alleviates doxorubicin-induced heart failure and excessive accumulation of ceramides. Front Cardiovasc Med. 2021;8:732554. doi: 10.3389/fcvm.2021.732554 34869633 PMC8639694

[6] Osataphan N, Phrommintikul A, Chattipakorn SC, Chattipakorn N. Effects of doxorubicin-induced cardiotoxicity on cardiac mitochondrial dynamics and mitochondrial function: insights for future interventions. J Cell Mol Med. 2020;24(12):6534–57. doi: 10.1111/jcmm.15305 32336039 PMC7299722

[7] Choksey A, Timm KN. Cancer therapy-induced cardiotoxicity-a metabolic perspective on pathogenesis, diagnosis and therapy. Int J Mol Sci. 2021;23(1):441. doi: 10.3390/ijms23010441 35008867 PMC8745714

[8] Menon D, Kadiu G, Sanil Y, Aggarwal S. Anthracycline treatment and left atrial function in children: a Real-Time 3-Dimensional Echocardiographic Study. Pediatr Cardiol. 2022;43(3):645–54. doi: 10.1007/s00246-021-02769-w 34787697

[9] Li VW-Y, So EK-F, Wong WH-S, Cheung Y-F. Myocardial deformation imaging by speckle-tracking echocardiography for assessment of cardiotoxicity in children during and after chemotherapy: a systematic review and meta-analysis. J Am Soc Echocardiogr. 2022;35(6):629–56. doi: 10.1016/j.echo.2022.01.017 35149208

[10] Chaulin AM. The essential strategies to mitigate cardiotoxicity caused by doxorubicin. Life (Basel). 2023;13(11):2148. doi: 10.3390/life13112148 38004288 PMC10672543

[11] Huang J, Wu R, Chen L, Yang Z, Yan D, Li M. Understanding anthracycline cardiotoxicity from mitochondrial aspect. Front Pharmacol. 2022;13:811406. doi: 10.3389/fphar.2022.811406 35211017 PMC8861498

[12] van Dalen EC, Caron HN, Dickinson HO, Kremer LC. Cardioprotective interventions for cancer patients receiving anthracyclines. Cochrane Database Syst Rev. 2011;2011(6):CD003917. doi: 10.1002/14651858.CD003917.pub4 21678342 PMC6457676

[13] Reichardt P, Tabone M-D, Mora J, Morland B, Jones RL. Risk-benefit of dexrazoxane for preventing anthracycline-related cardiotoxicity: re-evaluating the European labeling. Future Oncol. 2018;14(25):2663–76. doi: 10.2217/fon-2018-0210 29747541

[14] Songbo M, Lang H, Xinyong C, Bin X, Ping Z, Liang S. Oxidative stress injury in doxorubicin-induced cardiotoxicity. Toxicol Lett. 2019;307:41–8. doi: 10.1016/j.toxlet.2019.02.013 30817977

[15] Kong C-Y, Guo Z, Song P, Zhang X, Yuan Y-P, Teng T, et al. Underlying the mechanisms of doxorubicin-induced acute cardiotoxicity: oxidative stress and cell death. Int J Biol Sci. 2022;18(2):760–70. doi: 10.7150/ijbs.65258 35002523 PMC8741835

[16] do Nascimento TC, Cazarin CBB, Maróstica MR Jr, Mercadante AZ, Jacob-Lopes E, Zepka LQ. Microalgae carotenoids intake: influence on cholesterol levels, lipid peroxidation and antioxidant enzymes. Food Res Int. 2020;128:108770. doi: 10.1016/j.foodres.2019.108770 31955741

[17] Mao M, Zheng W, Deng B, Wang Y, Zhou D, Shen L, et al. Cinnamaldehyde alleviates doxorubicin-induced cardiotoxicity by decreasing oxidative stress and ferroptosis in cardiomyocytes. PLoS One. 2023;18(10):e0292124. doi: 10.1371/journal.pone.0292124 37824478 PMC10569550

[18] Zhao L, Qi Y, Xu L, Tao X, Han X, Yin L, et al. MicroRNA-140-5p aggravates doxorubicin-induced cardiotoxicity by promoting myocardial oxidative stress via targeting Nrf2 and Sirt2. Redox Biol. 2018;15:284–96. doi: 10.1016/j.redox.2017.12.013 29304479 PMC5975069

[19] Cheng Y, Wu X, Nie X, Wu Y, Zhang C, Lee SM-Y, et al. Natural compound glycyrrhetinic acid protects against doxorubicin-induced cardiotoxicity by activating the Nrf2/HO-1 signaling pathway. Phytomedicine. 2022;106:154407. doi: 10.1016/j.phymed.2022.154407 36070662

[20] Zhan G, Wei T, Xie H, Xie X, Hu J, Tang H, et al. Autophagy inhibition mediated by trillin promotes apoptosis in hepatocellular carcinoma cells via activation of mTOR/STAT3 signaling. Naunyn Schmiedebergs Arch Pharmacol. 2024;397(3):1575–87. doi: 10.1007/s00210-023-02700-5 37676495

[21] Dai W, Liu K, Li R, Cao Y, Shen M, Tao J, et al. Trillin inhibits myoblast differentiation via increasing autophagy. Phytomedicine. 2022;99:153962. doi: 10.1016/j.phymed.2022.153962 35172256

[22] Passos FRS, Araújo-Filho HG, Monteiro BS, Shanmugam S, Araújo AA de S, Almeida JRG da S, et al. Anti-inflammatory and modulatory effects of steroidal saponins and sapogenins on cytokines: a review of pre-clinical research. Phytomedicine. 2022;96:153842. doi: 10.1016/j.phymed.2021.153842 34952766

[23] Jiang W, Luo F, Lu Q, Liu J, Li P, Wang X, et al. The protective effect of Trillin LPS-induced acute lung injury by the regulations of inflammation and oxidative state. Chem Biol Interact. 2016;243:127–34. doi: 10.1016/j.cbi.2015.09.010 26363199

[24] Du J, Chen X, Wang F, Qin X, Zhao F, Tang X. Effect of trillin on oxidative stress and nuclear factor E2-related factor 2/antioxidant response element path‐way in rats after spinal cord injury. Chinese Journal of Rehabilitation Theory and Practice. 2019;25(10):1140–5. doi: insert_doi_here

[25] Xia D, Zhang T, Chen Y, Wang G, Yang L, Zhao F, et al. Effect of total saponins from Trillium tschonoskii Maxim on D-galactose-induced mitophagy in myocardial tissue in SD rats. Chinese Journal of Pharmacology and Toxicology. 2021;35(08):582–7.

[26] Zheng Q, Wang H, Hou W, Zhang Y. Use of Anti-angiogenic drugs potentially associated with an increase on serum AST, LDH, CK, and CK-MB activities in patients with cancer: a Retrospective Study. Front Cardiovasc Med. 2021;8:755191. doi: 10.3389/fcvm.2021.755191 34926609 PMC8674572

[27] Bellezza I, Giambanco I, Minelli A, Donato R. Nrf2-Keap1 signaling in oxidative and reductive stress. Biochim Biophys Acta Mol Cell Res. 2018;1865(5):721–33. doi: 10.1016/j.bbamcr.2018.02.010 29499228

[28] Luo W, Zou X, Wang Y, Dong Z, Weng X, Pei Z, et al. Critical role of the cGAS-STING pathway in doxorubicin-induced cardiotoxicity. Circ Res. 2023;132(11):e223–42. doi: 10.1161/CIRCRESAHA.122.321587 37154056

[29] Reis-Mendes A, Ferreira M, Duarte JA, Duarte-Araújo M, Remião F, Carvalho F, et al. The role of inflammation and antioxidant defenses in the cardiotoxicity of doxorubicin in elderly CD-1 male mice. Arch Toxicol. 2023;97(12):3163–77. doi: 10.1007/s00204-023-03586-1 37676301 PMC10567829

[30] Zhang J, Li W, Xue S, Gao P, Wang H, Chen H, et al. Qishen granule attenuates doxorubicin-induced cardiotoxicity by protecting mitochondrial function and reducing oxidative stress through regulation of Sirtuin3. J Ethnopharmacol. 2024;319(Pt 1):117134. doi: 10.1016/j.jep.2023.117134 37714227

[31] Mukta MM, Hossain MJ, Akter M, Banik B, Mithun MMZ, Sarwar S, et al. Cardioprotection of Water Spinach (Ipomoea aquatica), Wood Apple (Limonia acidissima) and Linseed (Linum usitatissimum L.) on Doxorubicin-Induced Cardiotoxicity and Oxidative Stress in Rat Model. Nutr Metab Insights. 2023;16:11786388231212116. doi: 10.1177/11786388231212116 38024869 PMC10666662

[32] Yarmohammadi F, Rezaee R, Karimi G. Natural compounds against doxorubicin-induced cardiotoxicity: a review on the involvement of Nrf2/ARE signaling pathway. Phytother Res. 2021;35(3):1163–75. doi: 10.1002/ptr.6882 32985744

[33] Li D, Zhang W, Fu H, Wang X, Tang Y, Huang C. DL-3-n-butylphthalide attenuates doxorubicin-induced acute cardiotoxicity via Nrf2/HO-1 signaling pathway. Heliyon. 2024;10(5):e27644. doi: 10.1016/j.heliyon.2024.e27644 38486757 PMC10938138

[34] Wu Z, Zai W, Chen W, Han Y, Jin X, Liu H. Curdione ameliorated doxorubicin-induced cardiotoxicity through suppressing oxidative stress and activating Nrf2/HO-1 pathway. J Cardiovasc Pharmacol. 2019;74(2):118–27. doi: 10.1097/FJC.0000000000000692 31356549

[35] Fang G, Li X, Yang F, Huang T, Qiu C, Peng K, et al. Galangin attenuates doxorubicin-induced cardiotoxicity via activating nuclear factor erythroid 2-related factor 2/heme oxygenase 1 signaling pathway to suppress oxidative stress and inflammation. Phytother Res. 2023;37(12):5854–70. doi: 10.1002/ptr.7991 37655750

[36] Reagan-Shaw S, Nihal M, Ahmad N. Dose translation from animal to human studies revisited. FASEB J. 2008;22(3):659–61. doi: 10.1096/fj.07-9574LSF 17942826

[37] Zhan G, Hu J, Xiao B, Wang X, Yang Z, Yang G, et al. Trillin prevents proliferation and induces apoptosis through inhibiting STAT3 nuclear translocation in hepatoma carcinoma cells. Med Oncol. 2020;37(5):44. doi: 10.1007/s12032-020-01369-7 32270306

